# Rare Cause of Skull Base Osteomyelitis: A Challenging Diagnosis and Management With a Literature Review

**DOI:** 10.7759/cureus.45838

**Published:** 2023-09-24

**Authors:** Syed Zohaib Maroof Hussain, Ibrahim I Haq, Zaki Arshad, Ayomide Ekunola, Sudip Das

**Affiliations:** 1 Ear, Nose and Throat (ENT), Leicester Royal Infirmary, Leicester, GBR; 2 Ear, Nose and Throat (ENT), University Hospitals of Leicester NHS (National Health Service) Trust, Leicester, GBR; 3 Trauma and Orthopaedic Surgery, Cambridge University School of Clinical Medicine, Cambridge, GBR

**Keywords:** skull base osteomyelitis, scedosporium apiospermum, malignant otitis externa, necrotizing otitis externa, fungal ear infection

## Abstract

This is a case of skull base osteomyelitis (SBO) caused by a rare fungal species, Scedosporium apiospermum. This is a clinical case report with a review of the literature. SBO is a potentially life-threatening infection of the temporal bone. The patient presented to our hospital with a two-month history of left otalgia, otorrhea and reduced hearing, after failed initial intravenous antibiotic therapy. Thorough examination and further investigation confirmed the diagnosis of SBO caused by a rare fungal species, S. apiospermum. The patient was subsequently started on a long-term course of antifungals which led to an improvement of symptoms. This case highlights the importance of early recognition and considering early antifungal treatment in patients with persistent otalgia and otorrhea, particularly in those who have failed to respond to intravenous antibiotics. Further research is needed to better understand the optimal timing and duration of antifungal therapy in these patients.

## Introduction

Necrotizing otitis externa (NOE), also referred to as skull base osteomyelitis (SBO) secondary to otitis externa, is a potentially life-threatening infection of the temporal bone [1]. The most common organism isolated in NOE is the bacterium Pseudomonas aeruginosa [2]. However, in rare cases, fungal species have also been found to be responsible [2]. Fungal NOE is predominantly an infection that affects immunocompromised patients and has been found in the literature to lead to disseminated infection and death [3]. Common fungal pathogens are Aspergillus and Candida species [4]. Although rare, Scedosporium apiospermum (S. apiospermum) is also responsible for fungal NOE, which is very difficult to diagnose and treat [4]. 

S. apiospermum is a filamentous fungus usually found in the environment and is commonly found as a commensal of the external ear [3]. NOE is a non-neoplastic, infective process affecting the external auditory canal (EAC) with subsequent invasion of the base of the skull [3]. SBO secondary to fungal species, particularly with S. apiospermum, can lead to fatal complications such as mycotic aneurysms and cerebral infractions [3,5]. In addition, delay in early recognition and treatment can also lead to cranial nerve palsies, intracranial abscesses, and sinus thrombosis [6]. Johnson et al. reported that mortality in SBO is high and can reach up to 10 to 20% [7]. 

To the best of our knowledge, there are only a few case reports of fungal NOE caused by S. apiospermum [3,6].

Here, we describe a case of an elderly male with signs and symptoms of ear infection, who was subsequently diagnosed with fungal NOE caused by a rare fungal species, S. apiospermum. The aim of this case is to highlight the importance of systemic examination and complete workup in dealing with patients with chronic ear infections, particularly resistant to antibiotics. 

## Case presentation

An 81-year-old gentleman with poorly controlled diabetes presented to our hospital with a two-month history of left otalgia, otorrhea and reduced hearing. The patient had a failed treatment (intravenous (IV) antibiotics of co-amoxiclav) in India for four weeks.

Comprehensive ear, nose and throat (ENT) examination was performed, which showed polyps in the left EAC, granulation tissue, and inflamed canal, and increased serous discharge was present. No signs of cranial nerve palsy or mastoiditis were present. 

His blood tests on admission showed mildly raised inflammatory markers. His initial HbA1c was 73mmol/mol as shown in Table 1. Ear swabs were taken as well, which grew E. coli. 

**Table 1:** Laboratory Investigations

Investigations	Patient’s values	Reference range
White cell count	12.0x 10*9/L	4.0 – 11.0 x 10*9/L
Neutrophils	9.47x 10*9/L	1.5 - 8.0 x 10*9/L
C-reactive protein	19 mg/L	<5 mg/L
HbA1c	73 mmol/mol	20-41 mmol/mol

**Figure 1:**
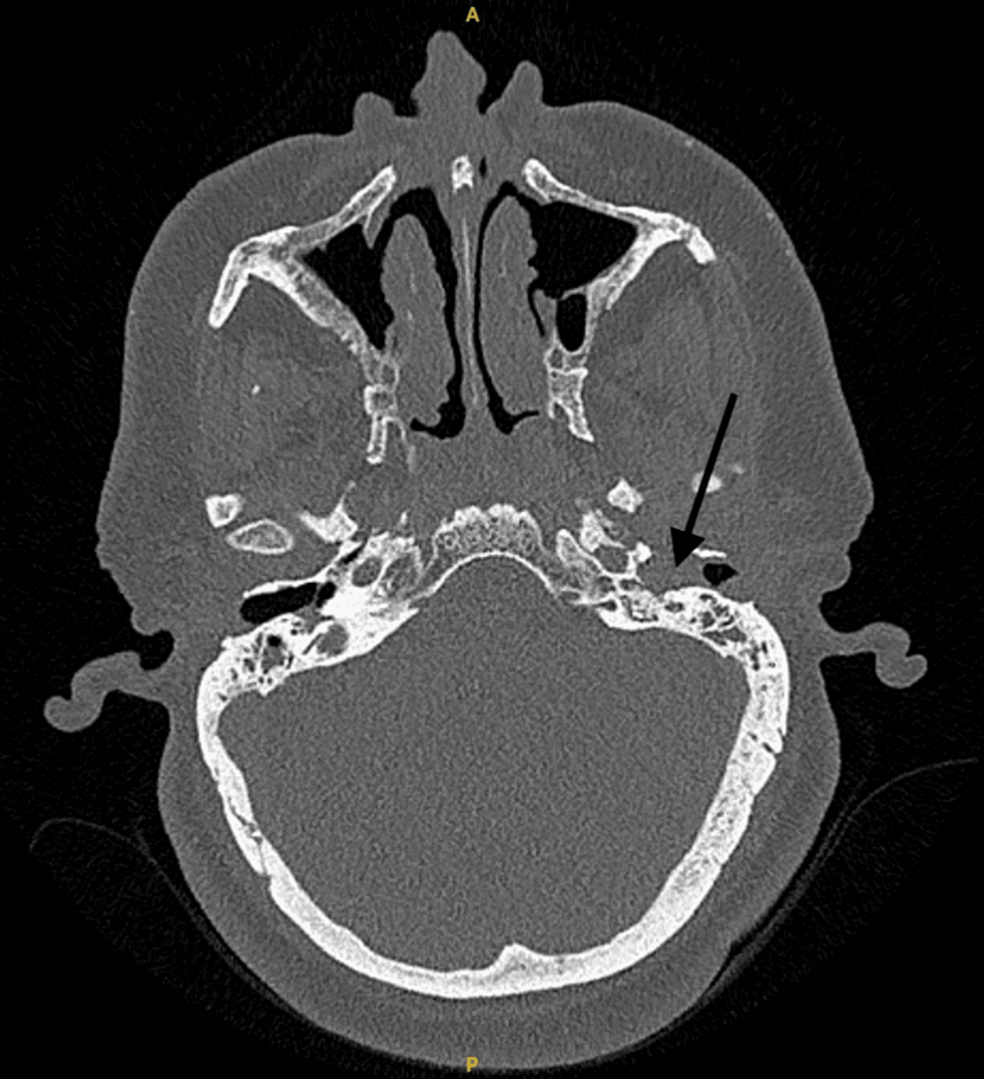
Axial CT scan showing erosion of left EAC and left EAC polyp (indicated by an arrow). EAC: External auditory canal

Computerized tomography (CT) scan of the petrous bone was performed, which revealed left NOE (Figure 1). 

**Figure 2:**
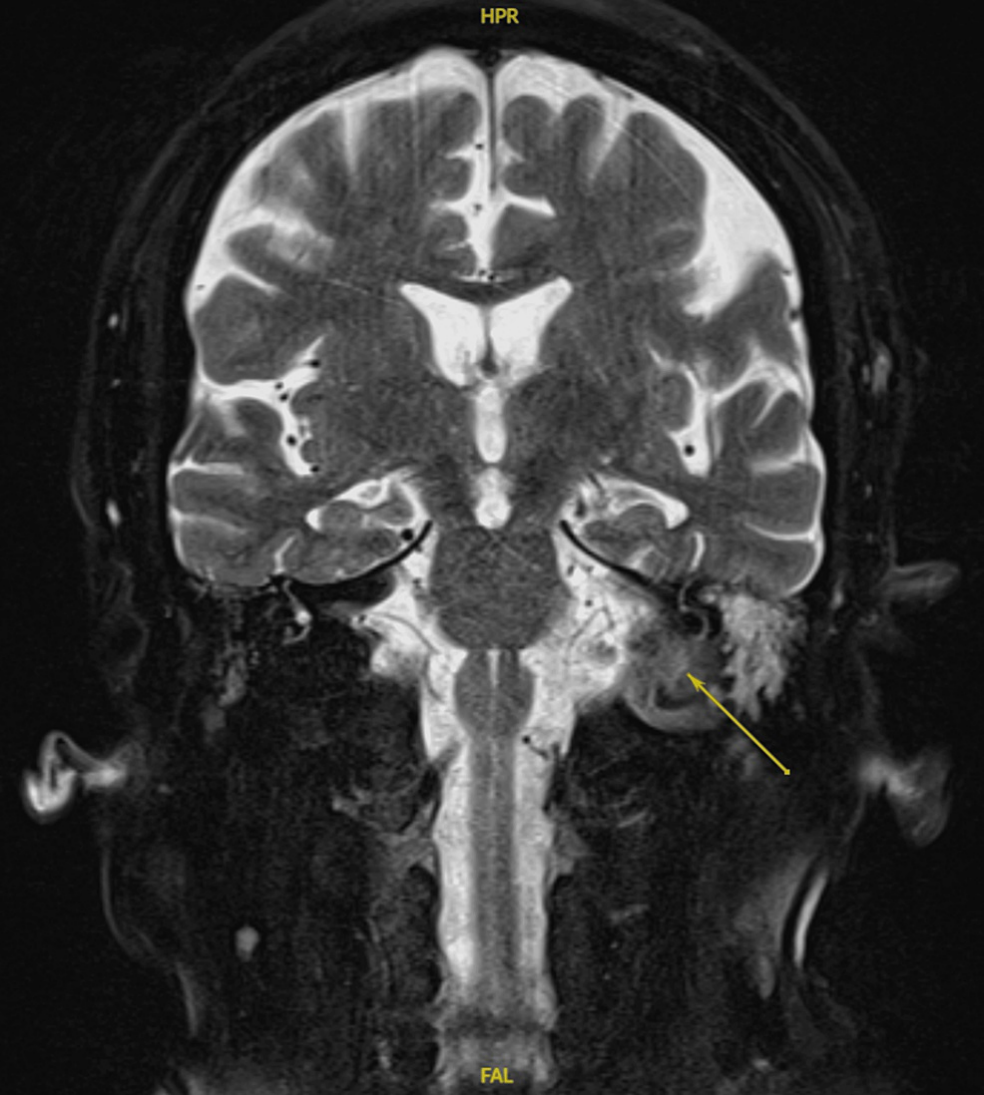
Coronal MRI IAM showing marked soft tissue thickening, oedema in the left EAC, retrocondylar space, parapharyngeal space and carotid space (indicated by an arrow). IAM: Internal auditory meatus; EAC: external auditory canal

Subsequently, a magnetic resonance imaging (MRI) scan of the internal auditory meatus (IAM) was carried out. This showed marked soft tissue thickening, oedema in the left EAC, retrocondylar space, parapharyngeal space and carotid space, which was suggestive of NOE without any evidence of abscess formation or intra-axial extension (Figure 2). 

The presenting symptoms and clinical findings of the patient in question gave rise to a differential diagnosis that included NOE, acute or chronic otitis externa, squamous cell carcinoma, cholesteatoma or an infected ear furuncle. Due to the increased inflammatory markers as well as the primary clinical complaint of pain and noting the medical background, the patient was empirically treated for NOE.

This patient was admitted under ENT for treatment as per the local NOE protocol. He was started on IV antibiotics as per the hospital antimicrobial guidelines for NOE, IV piperacillin and tazobactam (Tazocin), and ciprofloxacin ear drops. Despite focused antimicrobial therapy, his symptoms did not improve. The patient was discussed at a multidisciplinary team (MDT) meeting, and the outcome was to switch to IV ceftazidime and vancomycin. In addition, the patient was started on a buprenorphine (Butrans) 5 microgram patch, as well as sublingual buprenorphine to be taken as required for pain management. 

He was discharged home with a peripherally inserted central catheter line for long-term IV ceftazidime and oral doxycycline. The patient was followed regularly in the clinic for microsuctioning. 

At five weeks, the patient reported progression in symptoms along with facial weakness. Examination showed recurrence of a polyp in the left EAC and cranial nerve VII palsy as well. He was then readmitted for pain management and IV antibiotics. Ear swabs and biopsy from the polyp were sent to the lab. The case was discussed with a microbiologist, and they updated us that the patient's ear swab grew S. apiospermum in both samples. The microbiologist advised to commence an antifungal (voriconazole) based on the swab results. 

A repeated MRI diffusion-weighted imaging revealed mild progression of the inflammatory changes extending into the nasopharynx (not shown). 

This patient was rediscussed in the MDT meeting in light of the new investigation findings, and he was diagnosed with SBO caused by a rare fungal infection. Based on the MDT discussion, tigecycline and ciprofloxacin ear drops were added in addition to voriconazole. In addition, the patient had the removal of dead bone and remnants of the polyp under general anaesthesia. As a result, the patient started to improve. 

The patient was then discharged from the hospital on oral antifungals, regular analgesia, and regular follow-up. At the most recent follow-up, this patient had improved clinically with complete resolution of facial nerve palsy and no tragal or mastoid tenderness. He continues to have follow-up every two weeks with the NOE nurse practitioners for symptom monitoring and regular microsuction. He has also been followed up with his general practitioner for better diabetic control.

## Discussion

Aspergillus spp is a common cause of fungal NOE [8]. However, NOE due to S. apiospermum is exceptionally rare and challenging to diagnose [4,6]. Fungal hyphae of different fungal species cannot be differentiated initially in microscopic examination [3]. Therefore, it delays the diagnosis and appropriate treatment as well. As described in our case report, the patient's ear swab initially showed E. coli and no fungal growth was noted. Similarly, McLaren et al. reported a case of NOE caused by S. apiospermum. In their case report, they had difficulty in isolating a pathogen, which ultimately led to a delay in starting antifungals [6].

Similarly, Martin et al. reported in their study that fungal pathogens are usually diagnosed after the failure of multiple oral and topical antibacterial medications [9]. Our patient had four weeks of unsuccessful IV antibiotics and topical treatment in India. Later, we commenced on different antibiotics after discussing with a microbiologist. However, the patient did not show any improvement; in fact, he developed further complications at follow-up. This raised the suspicion of fungal cause, and this was confirmed on ear swabs. Hence, he was started on voriconazole. 

S. apiospermum is a highly resistant pathogen, [7] and the majority of cases involving S. apiospermum have been reported in patients who are immunosuppressed due to comorbidities, medical therapy, or prior organ transplantation [10]. Similarly, our patient was diabetic, and his blood sugars were poorly controlled. Therefore, we recommend close monitoring of blood sugars and a thorough diabetic review as well. 

Voriconazole is a safe and effective antifungal for S. apiospermum [4]. Previously published case reports have shown effective results as well [3,6]. S. apiospermum is usually resistant to amphotericin B. However, voriconazole has shown excellent results against S. apiospermum [11]. However, there is no clear guidance regarding the length of antifungal treatment. 

Like in our case, the patient did show improvement after we commenced him on voriconazole. It is essential to start to recognise failure of antibiotic treatment at an early stage, and clinicians should have a lower threshold for starting antifungal therapy in patients who are not responding to IV antibiotics. 

Furthermore, regular ear examination, aural toilets and, in some cases, wound debridement should be done if SBO is very severe to prevent fatal complications. 

It is also important to do multiple swabs, especially in patients who do not show any improvement or limited improvement against antibiotic therapy. In addition, CT and MRI scanning should be done to assess the extent of the disease. Traditionally clinicians use CT imaging alone for rapid initial assessment of the extension of disease and often use MRI later on to reveal the extent of soft tissue involvement, bone marrow infiltration, and intracranial involvement. In some cases, the use of technetium Tc-99 m and gallium scanning have also been reported. Technetium and gallium scans can be useful for serial follow-up imaging to look for disease resolution or progression [12,13]. A combination of various imaging modalities is utilized in clinical practice to diagnose and assess the severity of disease. CT, MRI and Tc-99 imaging may have limited use when following up with patients in an outpatient setting as changes in these scanning modalities can persist despite successful treatment [13]. Therefore, we also recommend assessing improvement clinically along with radiological imaging. 

## Conclusions

This case highlights the importance of considering fungal infections in patients with persistent otalgia and otorrhea, particularly in those who have failed to respond to IV antibiotics. Voriconazole should be considered as a treatment option in such cases, as it has been shown to be effective in treating fungal NOE. Further research is needed to better understand the optimal timing and duration of antifungal therapy in these patients.
